# Improving total bone marrow and lymphoid irradiation: feasibility of intensity-modulated proton therapy (IMPT) and dosimetric comparison with helical tomotherapy (HT)

**DOI:** 10.1186/s13014-024-02572-w

**Published:** 2025-05-21

**Authors:** Dayananda Shamurailatpam Sharma, Arjunan Manikandan, Gaganpreet Singh, Sanjib Gayen, Sham Sundar, Aishwarya G, Rangasamy S, Mahammood Suhail, Rajesh S, Ganapathy Krishnan, Revathi Raj, Srinivas Chilukuri, Jose Easow, Rakesh Jalali

**Affiliations:** 1 Department of Medical Physics, Apollo Proton Cancer Centre, Chennai, Tamil Nadu India; 2 Department of Radiation Oncology, Apollo Proton Cancer Centre, Chennai, Tamil Nadu India; 3 Department of Pediatric Hematology, Oncology, Blood and Marrow Transplantation, Apollo Cancer Centre, Chennai, Tamil Nadu India; 4 Department of Medical Oncology, Hematology, BMT and Cellular Therapy, Apollo Proton Cancer Centre, Chennai, Tamil Nadu India

**Keywords:** IMPT, TMI, TMLI, TBI, Dosimetry, Proton beam therapy, HSCT, PSQA

## Abstract

**Background and purpose:**

This study aims to develop and validate a novel whole-body intensity-modulated proton therapy (IMPT) approach for total marrow/lymphatic irradiation (TMI/TMLI) and compare its efficacy to helical tomotherapy (HT).

**Materials and methods:**

Whole-body IMPT plans were designed using five isocenters and fifteen fields for adult and three isocenters and eight fields for pediatric. Overlapping sub-PTVs were optimized to ensure robust and homogeneous dose distribution across consecutive isocenters and across CT datasets. Dosimetric benefits were compared to HT, safety and accuracy were verified using an in-house algorithm and planar dosage measurement.

**Results:**

The average cranio-caudal and lateral whole-body PTV dimensions were 169.24 cm and 46.41 cm for five adults; 95.2 cm and 28.86 cm for one pediatric patient. IMPT plans provided adequate and homogeneous dose to all sub-PTVs, upper and lower body PTVs, with mean D95% ≥ 11.4 GyRBE. In comparison to HT, IMPT plans reduced mean integral dosage to normal tissue by 38%, OARs by a factor of 1.32 to 3.94, and V107% by 520.97 cc for sub-PTVs and 1166 cc for upper-body PTVs. For 38 pairs of planned and measured dosage planes at three depths, the average (± SD) gamma value was 96.77% (± 2.45%). Radiation ON time of 76 and 28 min for the tallest adult and pediatric patient plan was almost double HT plan of 39.9 and 14.1 min.

**Conclusion:**

The presented whole-body IMPT approach for TMI/TMLI patients of any physical build-up is dosimetrically superior, safe and feasible to implement. Nevertheless, detailed robustness evaluation and cost-benefit analyses should guide its clinical implementation.

## Introduction

Total body irradiation (TBI) as a myeloablative conditioning regimen before allogeneic hematopoietic stem-cell transplantation (HSCT) improves outcomes for myeloid and lymphoid leukemia [1–4]. Despite its efficacy, the adoption of TBI has declined due to concerns over radiation-induced toxicity. Selective irradiation of total marrow (TMI) and lymphatics (TMLI) reduces exposure to organs at risk (OARs) compared to TBI, thereby reducing radiation-induced toxicity while maintaining disease control [5–8]. This advancement presents new avenues for exploring TMI/TMLI’s potential in enhancing disease control in first-remission patients, escalating doses for high-risk patients, employing reduced-intensity conditioning for elderly and comorbid patients, and refining regimens to minimize graft-versus-host disease post-HSCT [9, 10].

Presently, advanced radiotherapy techniques utilizing high-energy X-rays, such as helical tomotherapy (HT), intensity-modulated radiotherapy (IMRT), and volumetric modulated arc therapy (VMAT), are employed for TMI/TMLI procedures [5–8]. While these techniques offer advantages, the unique physical properties of proton Bragg peak, characterized by no exit dose and higher relative radiobiological effectiveness (RBE) compared to X-rays, hold promise for further minimizing doses to OARs while maintaining the target dose. However, the application of proton therapy for TMI/TMLI has been limited by several challenges, including restricted radiation field size, difficulties in achieving dose homogeneity in field abutment regions, limited beam delivery angles, constraints in treatment planning optimization algorithms, and the need for numerous custom beam apertures and tissue compensators for passive scattering techniques.

Recently, Zuro DM et al. [11] explored intensity modulated proton therapy (IMPT) for pediatric upper body marrow irradiation, excluding extremities like hands, humeri, ulnae, and radii, and assessed dosimetric feasibility with a maximum planning target volume (PTV) length of 88.13 cm. We have conducted a comprehensive investigation into the dosimetric feasibility of whole-body IMPT for adult TMI/TMLI. Furthermore, we evaluated the potential dosimetric advantages of this novel technique compared to HT technique. Finally, we assessed the safety, accuracy, and efficacy of the proposed technique within the proton beam therapy (PBT) system. To the best of our knowledge, this study marks the first comprehensive investigation of the potential of whole-body IMPT for adult TMI/TMLI.

## Materials and methods

This study utilized treatment planning datasets comprising computed tomography (CT) scans and contours from five adults and one pediatric patient who underwent image-guided TMI/TMLI on Radixact HT (Accuray Inc, US). The study received institutional ethics committee approval (APH-C-S-003/02–24). The CT-simulation procedure, target volume definition, HT treatment planning and delivery including image guidance protocol, and a novel patient-specific quality assurance (PSQA) method have been reported in the literature [12, 13]. In brief, the patients were positioned in a whole-body vacuum bag and underwent CT scans in head-first supine (HFS) and feet-first supine (FFS) orientations with an overlap at mid-thigh. The clinical target volume (CTV) encompassed the entire skeletal structure and brain for TMI, and additionally included the spleen and key lymphatic regions for TMLI. The skeleton CTV was auto-delineated using a Hounsfield Unit (HU) threshold on HFS and FFS scans, followed by manual editing. The CTV skeleton was divided into multiple segments: skull, chest, upper and lower limbs, vertebrae, and pelvis, to allow for differential PTV margins. The mandible, hyoid bone, patella, and larynx were excluded from the CTV. Isotropic PTV margins of 3 mm, 5 mm, 7 mm, 10 mm, and 5 mm were applied to the CTV regions of the skull, vertebrae, chest/spleen, upper limb (and lower limb for FFS scan), and pelvis (and scrotum), respectively [12]. The CTV for lymphatic regions included cervical, supraclavicular, mediastinal, axillary, complete para-aortic chain, external and internal iliac, and inguinal nodes. A uniform margin of 5 mm was applied to generate lymph nodal PTVs. The variable margins for various CTVs are based on our institutional safety margin guidelines for treating specific clinical sites [12], supported by evidence from the published literature [8]. The margin protocol adopted in this study has been developed and deemed sufficient for TMI/TLI employing HT [12] and the same have been adopted for IMPT planning. The eyes, lens, parotids, midline mucosa (oral, pharyngeal, laryngeal, and tracheal), esophagus, lungs, heart, liver, kidneys, bladder, and colon were identified as organs at risk (OARs).

### IMPT treatment planning

The IMPT treatment plans were created using RayStation (v12A, RaySearch Laboratories, Sweden) treatment planning system (TPS), clinically commissioned for Proteus PLUS (IBA, Louvain-La-Neuve, Belgium) proton therapy system (PTS) [14]. Proteus PLUS is an isochronous cyclotron-based image-guided PTS featuring 360° gantry rotation and a dedicated spot scanning nozzle. It modulates proton energy from 226.2 to 70.2 MeV (32 to 4.1 g/ cm^2^) with a spot size (sigma) of 3 to 6.7 mm in air and at isocenter. An add-on range shifter, having a water equivalent thickness of 4 g/cm^2^, reduced the proton range below 4.1 g/cm^2^ to treat superficial tumors. The proton spot can be scanned in either a hexagonal or square pattern to define a maximum field size of 35 × 40 cm^2^.

**Fig. 1:**
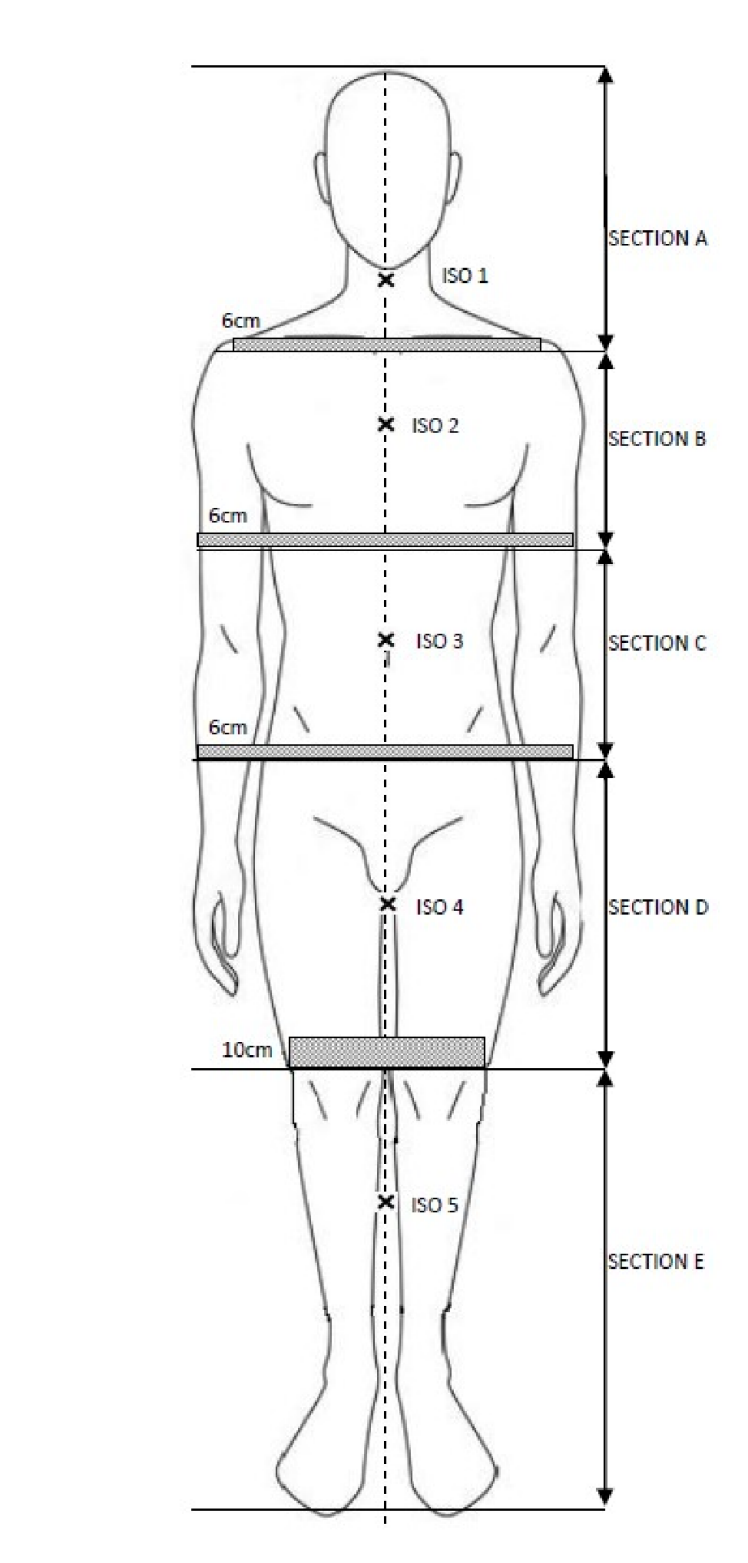
Schematic diagram describing spilt of upper and lower body PTV in to subsections with 6–7 cm overlap between two consecutive sections and 10 cm overlap between upper and lower body PTVs. It also indicate the approximate location of the iscenters represented by Iso 1, Iso 2….Iso 5 to create IMPT plan

Each patient’s IMPT plan was developed separately for the upper body using HFS-CT and the lower body using FFS-CT. For the purpose of treatment planning, upper body PTV was divided in to four sections with 6–7 cm overlap between two consecutive sections as shown in Fig. 1.

The inferior most 10 cm segment of the upper body PTV (section D, Fig. 1) was divided into 5 sub-volumes of similar length to establish a dose gradient that, when paired with the lower body IMPT plan, produced a homogeneous dosage in the overlap thigh region [15, 16]. The upper body IMPT plan comprised four strategically chosen isocenters, each with three fields except the second, which had four. Isocenters varied only in cranio-caudal coordinates, and optimal beam angles were determined based on target characteristics, dosimetric objectives, and implementation simplicity. The chosen beam geometry closely mimics a combination of regularly used beam geometries for various tumor sites, but is optimized for a minimum number and easier implementation. The lower body IMPT plan, created on FFS-CT, employed a single isocenter and two fields. The superior 10 cm of the lower body PTV was divided into five subsections (Section D/E, Fig. 1) to generate a complementary dose gradient that, when paired with the upper body plan, produces uniform dose distribution in the overlap thigh region. For the pediatric patient with short stature, an IMPT plan was developed using three isocenters with 8 beams on a single CT dataset, following a similar procedure as that of the adult upper body patients. Primary planning goals aimed to ensure 95% of PTVs received at least 95% (D95%) of the prescription dose of 12 GyRBE, with specified dose constraints for lung and OARs. The RayStation TPS’s Monte-Carlo (MC) algorithm was utilized for spot map optimization and volumetric dosage calculation.

### HT treatment planning

HT plans were produced using Precision TPS (iDMS v1.1.1.1, Accuray Inc, USA) separately for the upper and lower body employing HFS and FFS CT datasets, except for the pediatric patient, where a whole-body TMLI plan was created on a single CT dataset. For optimization, the entire target volume in HFS-CT was divided into PTV-upper and PTV-Junction, whereas FFS-CT was divided into PTV-lower and PTV-Junction. Similar to the IMPT plans for adult patients, the common PTV-Junction delineated in both CT series represents lower 10 cm of the PTV-upper and upper 10 cm of the PTV-lower (overlap region of sections D and E in Fig. 1). PTV-Junction were equally divided into five sub-volumes, each with 2 cm in length to establish a dose gradient in the overlapping 10 cm region, ensuring a uniform dose in the overlapping thigh region when combined [12]. Adult patients necessitated two isocenters, one for each upper and lower body, whereas the pediatric patient required only one. Two separate treatment plans were generated on each of the scans (HFS and FFS) to cover the entire target with a complementary dose gradient across the junction. The pitch ranged from 0.3 — 0.43, the field width was 5 cm, and the modulation factor was 2.5–3.5 for HFS orientation and 2.15–2.5 for FFS. The RayStation TPS was used to sum the plans generated on HFS-CT and FFS-CT. The details of the HT treatment plan are described elsewhere [12].

### Treatment plan evaluation and comparison with HT

Deformable image registration (DIR) between HFS-CT and FFS-CT scans was performed in RayStation TPS prior to treatment plan optimization, with manual adjustments as necessary. The DIR-derived anatomical deformation vectors were used to deform the dose from the FFS-CT onto the HFS-CT. The dose grids were then combined on the HFS-CT using the sumplan feature in RayStation, and the junction dose profile was evaluated for accuracy and consistency. The dosimetric outcomes of the treatment plans were evaluated qualitatively based on isodose distribution and quantitatively using standard dose volume indices. Optimal target coverage (D95%) was assessed for each PTV and for the upper and lower body PTVs. PTVs receiving more than 107% of the prescribed dose (V107%) were considered high-dose volumes. Mean doses to several OARs and the integral dose (ID_j_ = ρ_j_ V_j_ D_j_, where ρ_j_, V_j_ and D_j_ are the density, volume and mean dose of the sub-volume j) to normal tissue were also calculated. For this study, we assumed that the normal tissue had a density equal to its mean density and that all sub-volumes of the organ received mean dose D [15]. The dose volume indices derived from the IMPT plans were compared to the corresponding values obtained from the HT plans.

### Feasibility test of IMPT treatment plan delivery using an in-house script

The feasibility of the treatment plan for successful irradiation on the PTS was initially assessed using an in-house developed spot map analysis program written in Matrix Laboratory (MATLAB) (MathWorks Inc., MA, USA). The script processes each ion beam sequence in the treatment plan file, which contains multiple layers of scan spots across different gantry angles. For each layer, the scan spot position map is used to retrieve the X and Y spot coordinates. If any spot falls outside the defined limits, the coordinates are adjusted to the respective boundary values. The updated coordinates are then saved back to the layer’s data structure. Once the modifications are complete, the corrected DICOM-RT file is exported and re-imported into the TPS for verification of the dose distribution without optimization, and dosimetric outcomes are evaluated for clinical goal fulfillment. This process ensures that the PTS can deliver the plan.

### Validation of IMPT treatment plan delivery on PTS

The seamless integration and accuracy of dose delivery in the PTS were verified through direct measurement of dose planes at a collapsed gantry angle of 270° using a 2D ion chamber array (MatriXX-PT) immersed in a DegiPhan water phantom (IBA Dosimetry, Germany). Since the active detector area of MatriXX-PT (24 × 24 cm^2^) is smaller than the field size of the IMPT plan, each field was measured in two parts by shifting the detector ± 10 cm from the field center. Measurements were conducted at depths of 2.97 g/cm^2^, 5.01 g/cm^2^, and additionally at 8.99 g/cm^2^ for the Brain field. Agreement between the planned and measured dose distributions was quantified using gamma analysis set at a 3% dose difference at a 3 mm distance with a 10% dose threshold.

## Results

Table 1 summarizes the demographic data and target information of the patients. Among the six patients (three male, three female), five adults (median age: 36 years; range: 25–49 years) had an average (± SD) height of 166.16 cm (± 8.94 cm) and a mean (± SD) body mass index (BMI) of 22.85 (± 6.95) kg/m^2^. The lone pediatric patient (3 years/female) was 96 cm tall with a BMI of 15.08 kg/m^2^. The mean (± SD) extent of the whole-body PTV in the cranio-caudal and lateral directions was 169.24 cm (± 13.64 cm) and 46.41 cm (± 6.51 cm) for adults and 95.2 cm and 28.86 cm for the pediatric patient, respectively. Of these patients, five were planned for whole-body TMLI while one was planned for TMI. The mean (± SD) volume of the whole-body CTV and PTV was 9014.22 cc (± 3150.43 cc) and 17279.36 cc (± 4517.46 cc) for adults and 3106.3 cc and 5058.48 cc for the pediatric patient. The application of differential PTV margins for various CTVs resulted in an overall increase in the total volume by an average (± SD) value of 91.12% (± 24.25%).

**Table 1:** Patient demography, diagnosis, body mass index (BMI), and maximum extent of the PTV in cranio-caudal and lateral direction along with their total volume. CML: Cronic myloid lukemia, ALL: acute lymphoid lukemia, TMI: Total marrow irradiation, TMLI: Total marrow and lymphoid irradiation

Patient	Age	Sex	Diagnosis	Height (cm)	Weight (Kg)	BMI (Kg/m2)	Treatment Plan for	Maximum extent of whole body PTV		Volume (cc) of whole body		% Increase in volume
								Length (cm)	Width (cm)	CTV	PTV	
P1	49	M	CML	170.3	92.5	31.89	TMLI	173.60	53.80	10647.75	19845.72	86.38
P2	42	F	ALL	159.0	53.1	21.00	TMLI	154.40	46.40	8023.43	15358.92	91.43
P3	36	F	CML	160.0	54.0	21.09	TMI	164.30	48.66	5846.16	13719.43	134.67
P4	25	M	ALL	180.0	97.0	26.85	TMLI	182.39	47.22	13655.15	23953.49	75.42
P5	27	M	ALL	161.5	35	13.42	TMLI	163.49	35.96	6898.61	13519.25	95.97
P6	3	F	ALL	96.0	13.9	15.08	TMLI	95.20	28.86	3106.30	5058.48	62.85

**Fig. 2:**
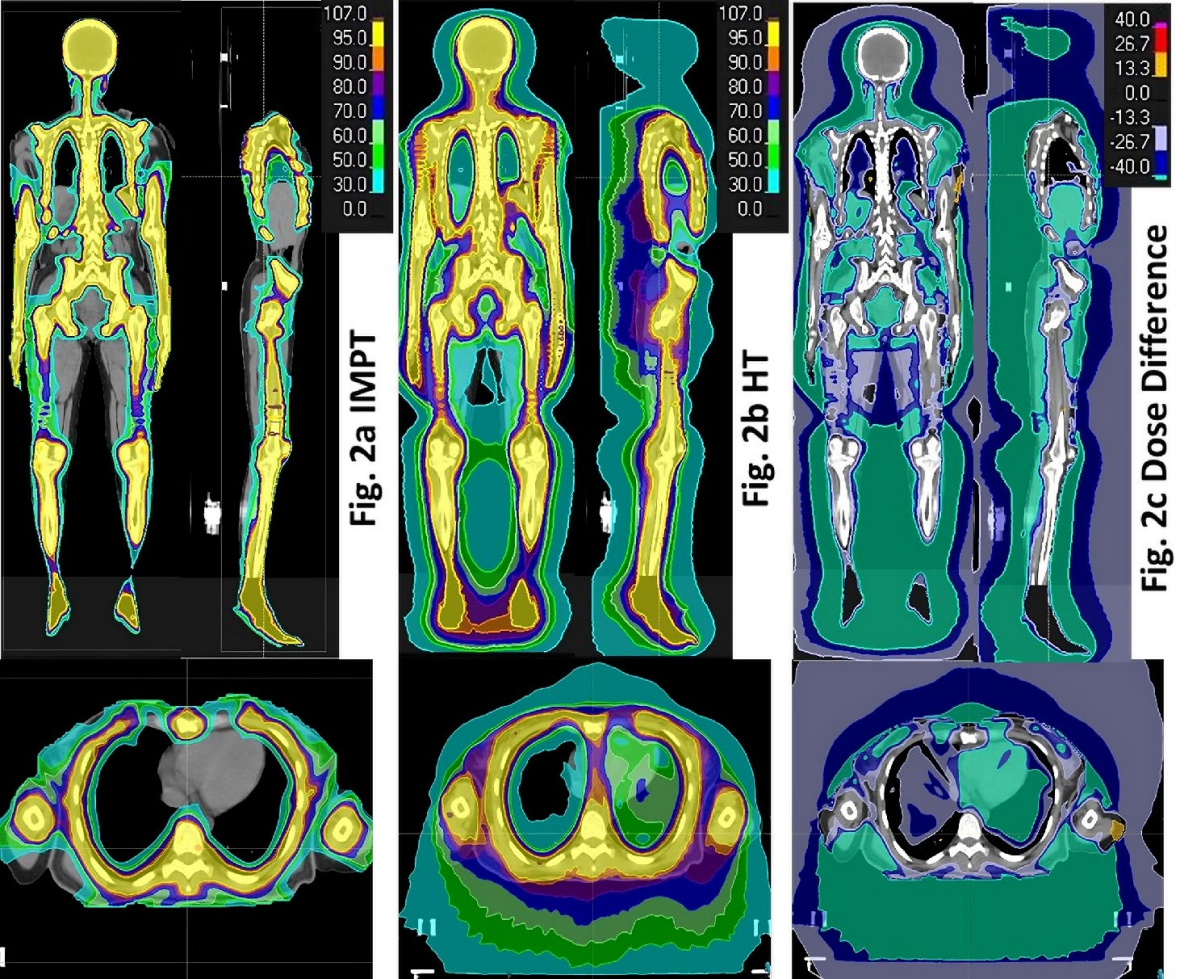
Composite dose distribution of upper and lower body treatment plans of a representative patient from IMPT (Fig. 2**a**), HT (Fig. 2**b**) and their differences (Fig. 2**c**). In comparison to HT plan, IMPT showed dose reduction of more than 40% in all normal tissue

The optimized gantry angles in degrees for each isocenter, starting from Sect. 1 (brain) to 4 (thigh) as shown in Fig. 1, are (60, 300, 180), (40, 120, 240, 320), (90, 180, 270), and (0, 120, 240) for the upper body, and (0, 180) for the lower body in adults, and (60, 300, 180), (50, 310, 180), and (0, 180) for the whole-body pediatric patient. The composite dose distribution from the upper and lower body IMPT plans of a representative adult patient (P1) is shown in Fig. 2a, while the corresponding dose distribution from the HT plan and their dose difference are shown in Fig. 2b and c, respectively.

The IMPT plan (Fig. 2a) demonstrates highly conformal and homogeneous dose distribution throughout the target volume, including overlapping regions of consecutive isocenters and CT datasets. It also shows a reduction of intermediate to low (< 50%) dose to all OARs and normal tissue compared to the HT plan (Fig. 2c).

**Fig. 3:**
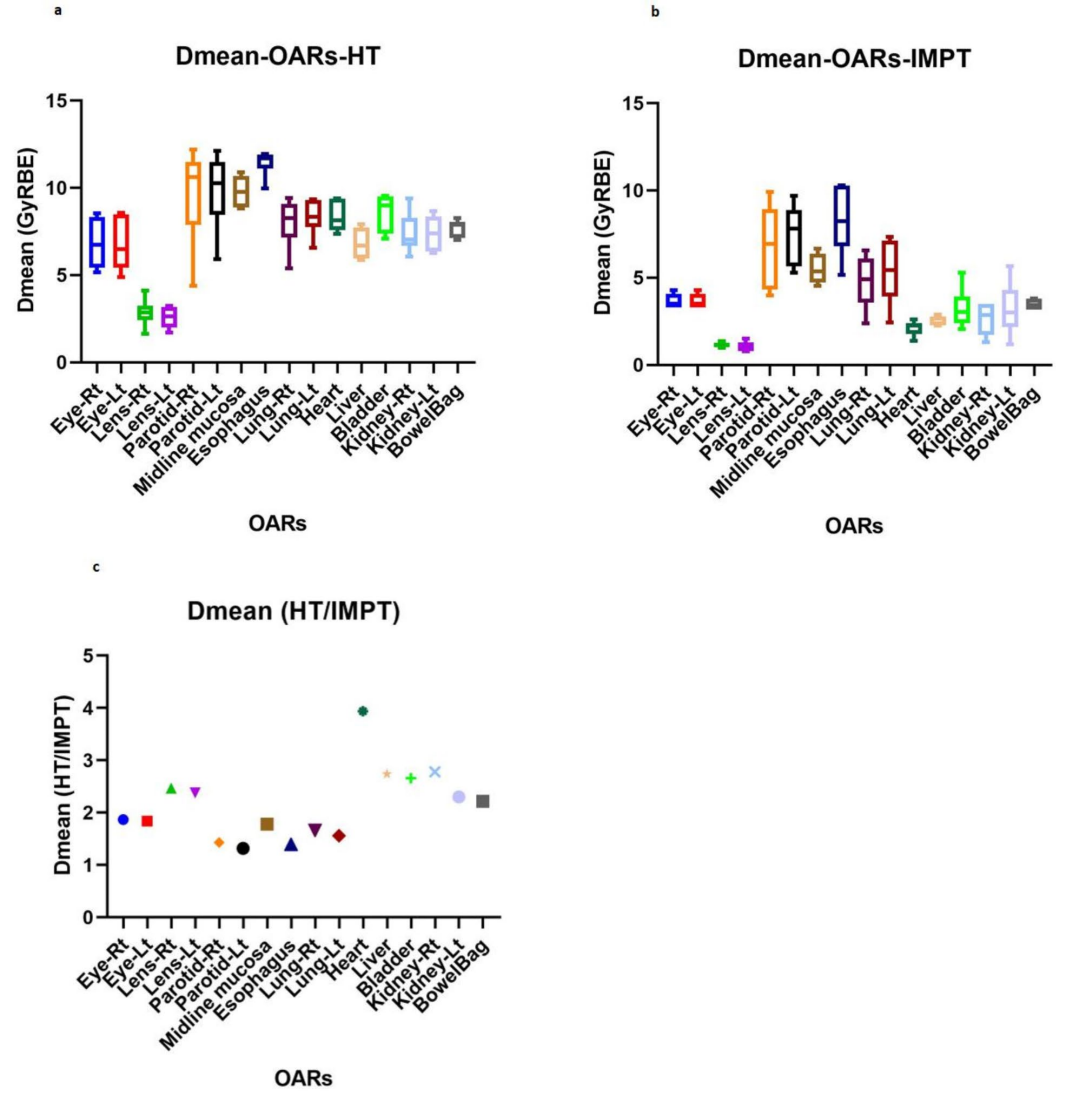
Box plot representing Dmean to several OARs of the 6 patients planned with (**a**) IMPT, (**b**) HT, and (**c**) ratio of HT by IMPT

The mean (± SD) values of D95% (GyRBE) for sub-PTVs (brain, chest, torso, upper limb, lymph node, spleen), upper and lower body PTVs for adults, and whole-body PTV for the pediatric patient, from IMPT and HT plans are presented in Table 2. IMPT plans provide adequate target coverage to all PTVs with a mean D95% ≥11.4 GyRBE. In comparison to the mean D95% of HT plans, the PTV-brain showed the least (0.04%) variation while the largest (4.06%) reduction was observed in PTV-chest, although it was comparable (0.55%) and (-0.42%) for the upper and lower body PTV. The maximum dose (D2%) to the upper body PTV averaged over the five adult patients was 12.62 (± 0.03) GyRBE for IMPT and 13.06 (± 0.37) GyRBE for HT plans. For similar target coverage, IMPT plans for adult patients showed a substantial reduction in high dose volume (V107%), as much as 520.97 cc for sub-PTVs (PTV-upper limb) and 1166.19 cc for upper body PTV compared to the HT plan. There is no appreciable difference in D95%, D2%, and V107% between IMPT and HT plans for the lone pediatric patient. In comparison to the HT plan, IMPT reduced the integral dose to normal tissue ranging from 31.45 to 48.69% (mean 38%). Figure 3 depicts the box plot of the mean dose to several OARs of the six patients planned with IMPT (Fig. 3a) and HT (Fig. 3b). The overall average Dmean from IMPT plans was less than 1.2 GyRBE for lenses; 3.5 GyRBE for eyes, heart, liver, kidneys, bladder, bowel beg; 5.5 GyRBE for midline mucosa, lungs; 7.5 GyRBE for parotids and 8.5 GyRBE for the esophagus. Compared to HT, IMPT plans decreased Dmean ranging from 1.32 times in the parotid to as high as 3.94 times in the heart (Fig. 3c).

**Table 2:** D95% (GyRBE) and V107% of different PTVs (brain, chest, torso, upper limb, lymph node, spleen) separately and the whole PTV (upper body) from IMPT and HT plans of the six patients

PTVs	Dose (GyRBE) to 95% volume of different PTVs					Volume (cc) of PTVs receiving > 107% of the prescribed dose				
	HT		IMPT		∆D95%	HT		IMPT		Absolute Diff (cc)
	Mean	±SD	Mean	±SD		Mean	±SD	Mean	±SD	
Brain	11.84	0.09	11.83	0.09	0.04	92.85	114.62	0.31	0.35	92.54
Chest	11.57	0.44	12.06	0.10	-4.06	159.71	147.45	1.11	2.19	158.59
Torso	11.78	0.23	11.59	0.26	1.67	270.24	272.51	0.65	1.32	269.59
Upper Limb	11.27	0.68	11.55	0.27	-2.44	522.81	413.56	1.84	3.22	520.97
Lymph Node	11.59	0.47	11.43	0.61	1.36	109.68	133.25	0.35	0.60	109.32
Spleen	11.71	0.36	11.42	0.43	2.57	4.13	7.43	0.13	0.22	4.00
Upper body	11.58	0.36	11.51	0.23	0.55	1170.66	1062.13	4.47	5.12	1166.19
Lower body	11.76	0.06	11.81	0.03	-0.42	168.39	154.49	0.55	0.92	167.84
Whole body (paediatric patient)	11.48	-	11.40	-	0.70	0.02	-	0.00	-	0.02

In one of the six patients enrolled in this study, the IMPT treatment plan was deemed undeliverable on the PTS. This occurrence prompted the development of an in-house spot map analysis algorithm to assess the validity of IMPT plans before exporting them to the PTS. The algorithm successfully identified spot positioning errors as the cause of plan delivery failure. Upon correction, the dose distribution from the original spot map and the new spot map generated by the in-house algorithm was comparable for this patient and was successfully deliverable on the PTS without any errors. The total radiation ON time for this IMPT plan, representing the largest whole-body PTV (P4), was 76 min, whereas it was 27.8 min for the pediatric patient.

**Fig. 4:**
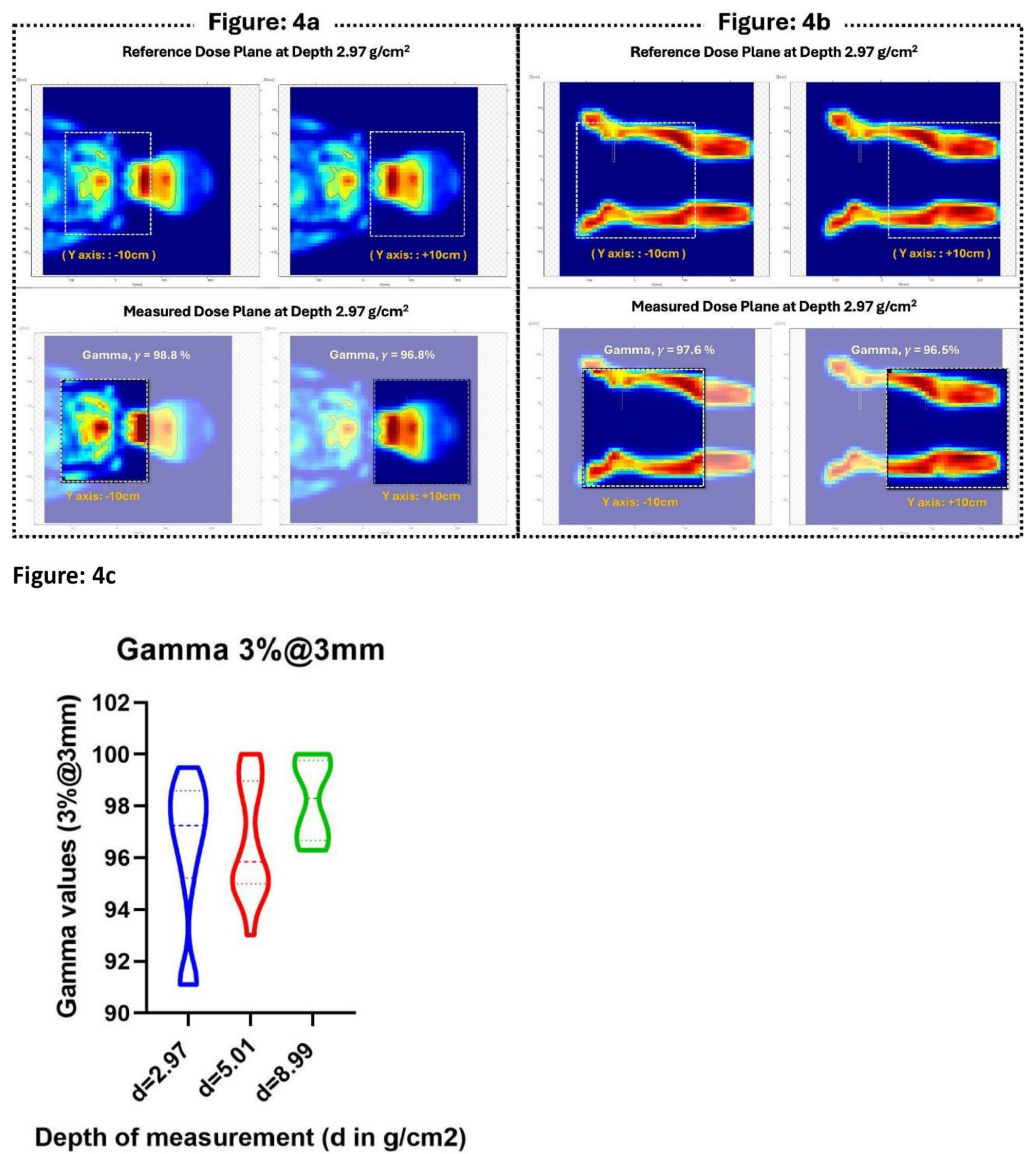
Comparison of the reference (top) and measured (bottom) dose plane at 2.97 cm depth for two posterior fields representing (**a**) head and neck and thorax (section A), (**b**) lower body PTV (section D & E). The rectangular dotted box represents area of comparison. For each field, comparison were carried out in two sections represented by Y = + 10 cm and Y=-10 cm. (**c**) Distribution of gamma values resulted from the comparison of 38 planned and measured dose planes at three depths for a representative patient (pediatric)

Figure 4 compares the reference (top) and measured (bottom) dose planes at 2.97 g/cm^2^ depth for two posterior fields: Fig. 4a) head and neck and thorax, Fig. 4b) lower body PTV from the full-body IMPT plan of the pediatric patient (P6).

The rectangular dotted box denotes the area of comparison. Each field was compared in two portions, Y = + 10 cm and Y=-10 cm from the field center. Figure 4c illustrates the gamma distribution from 38 pairs of dosage planes at three depths. In 16 planar dosage verifications at 2.97 g/cm^2^, gamma values were above 95%, with only three instances exceeding 91%, resulting in a mean (± SD) value of 96.37% (± 2.82%). At a depth of 5.01 g/cm^2^, 3 out of 16 verifications had gamma values below 95% but above 93%, resulting in a mean (± SD) of 96.63% (± 2.23%). All verifications showed gamma values over 95% (mean = 98.23%; SD = ± 1.57%) at 8.99 g/cm^2^.

## Discussion

Our study has established the feasibility, safety, and dosimetric accuracy of whole-body IMPT for TMI/TMLI. To the best of our knowledge, this investigation represent the first comprehensive examination of whole-body IMPT for TMI/TMLI treatment, encompassing a diverse patient dataset characterized by substantial variability in physical parameters such as PTV length, width, and body mass index. This variability allowed us to thoroughly assess the performance of the developed technique across a spectrum of clinical scenarios, providing valuable insights into its potential applicability in real-world settings.

In contrast to previous study [11] that primarily focused on upper body pediatric populations, our research extends the scope to include adult patients with varying physical attributes. Notably, we successfully demonstrated the feasibility of using IMPT to treat the entire bone marrow and lymphoid system in adult patients, including those with heights of up to 180 cm and widths up to 53.8 cm. Despite utilizing several isocenters, our approach is anticipated to be robust due to simultaneous optimization of overlapping regions between consecutive upper body PTV sections and between upper and lower body plans [15, 16]. The beam geometry, particularly in the thoracic region, is largely en-face beam, which may help to mitigate the range uncertainty associated with respiratory motion. Our planning method focusses on the simultaneous optimization of multiple PTVs, constrained primarily due to the utilization of two CT-datasets (HFS and FFS) to create whole body TMI/TMLI plan and also by the complexities associated with robust optimization for various sub-targets that may encounter distinct setup and range errors. Nevertheless, a comprehensive investigation into the effects of various set-up and range uncertainties, including those induced by respiration, on plan robustness is crucial for improving confidence in the development of a robust IMPT plan for TMI/TMLI. The limitation of this study is the lack of a comprehensive robustness evaluation of the IMPT plan, and the PTV margins are considered adequate to account for setup and range uncertainty. Our ongoing project aimed at addressing these challenges in a separate manuscript.

Our findings revealed significant advantages of IMPT over traditional HT plans, including improved target coverage, dosage homogeneity, and reduced OARs and integral dose and hot spots. This superior performance is particularly noteworthy given the challenges associated with extremity PTVs and larger patients, which often result in larger hot spot volumes in HT plans due to the thread effect. The substantial reduction in Dmean by a factor of 1.32 to 3.94 with IMPT could translate in to a significant decrease in the toxicity profile of several OARs. Additionally, the marked decrease in integral dose with IMPT may lower the risk of radiation-induced secondary cancers [17]. Furthermore, rigorous verification of planar doses demonstrated the reliability and precision of the whole-body IMPT plan for TMI/TMLI, with the majority of pairs exhibiting gamma values exceeding 95%. The PSQA took 2.5 h, however adult patients may take longer with more fields. Total radiation ON time of 76 min for tallest adult and 28 min for pediatric patient is almost double HT plan time of 39.9 min and 14.1 min for identical patient. However, radiation for 76 min will be administered in five intervals, each linked with one isocenter. The radiation ON time per isocenter for this patient varies between 10 – 22 min, depending on the number of fields and complexity of the proton spot map. Whereas, for the pediatric patient, radiation ON time per isocenter varies between 6 and 12 min.

Image guidance protocols may differ among institutes depending on the PT system (PTS) configuration. In the Proteus PLUS PTS, image guidance protocol for the treatment of multi-isocenter cranio-spinal axis irradiation (CSI) described by Sharma et al. [18] could be consider for the upper body TMI/TMLI. In summary, the setup could be verified by aligning the orthogonal radiograph of the second isocenter (thoracic region) with the corresponding digitally reconstructed radiograph (DRR). If the setup variation is within the institutional threshold, CBCT of the head and neck (first isocenter) can be performed without altering the patient’s position. The calculated six-dimensional correction vectors, are then applied simultaneously to all isocenters. Prior to treatment of subsequent isocenter, a verification scan either CBCT or planar X-ray can be performed to confirm the patient’s position accuracy following the setup corrections. Any subsequent shift detected in the cranio-caudal direction must be avoided or need justification.

Assuming similar patient set-up times across both modalities, the total treatment time largely depends on the image guidance protocol for set-up verification and radiation delivery time. For TMI/TMLI using HT, adult patients typically undergo 4–5 megavoltage computed tomography (MVCT) scans, each lasting 4–5 min. The average IN-TO-OUT time for an adult patient treated in HT is about 1.5 h [12]. Patient set-up can be verified using orthogonal radiographs, cone-beam computed tomography (CBCT), or a combination of both. CBCT image acquisition and verification typically take 3–4 min. During the first fraction, with 4–5 segment verifications, the set-up time may be reduced by up to 5 min compared to HT. Moreover, as the primary target in TMI/TMLI is bony structures, using only orthogonal radiographs may significantly reduce set-up verification time. Thus, the longer radiation ON time with IMPT can potentially be offset by shorter set-up verification times.

Ultimately, while the additional treatment time with IMPT is a manageable concern, the clinical decision to utilize IMPT over HT must be guided by a comprehensive assessment of the patient’s specific clinical needs, the dosimetric advantages of each modality, expected clinical benefit, and the capacity of the treatment facility. Further studies are warranted to explore strategies for reducing treatment time in IMPT, such as improved delivery techniques and the use of faster imaging and verification systems, which could enhance its practicality for whole-body irradiation.

## Conclusion

The presented whole-body IMPT approach for TMI/TMLI patients of any body habitus is dosimetrically superior compared to contemporary X-ray-based technique and is safe and feasible to implement. Nevertheless, a detailed robustness evaluation and cost-benefit analysis should guide its clinical implementation although it holds great promise for reducing radiation toxicity.

## Data Availability

No datasets were generated or analysed during the current study.

## Abbreviations

CT: Computed tomography
CTV: Clinical target volume
FFS: Feet-first supine
HFS: Head-first supine
HSCT: Hematopoietic stem-cell transplantation
IMPT: Intensity-modulated proton therapy
IMRT: Intensity-modulated radiotherapy
OARs: Organs at risk
PSQA: Patient-specific quality assurance
PTV: Planning target volume
RBE: Relative biological effectiveness
TMI: Total marrow irradiation
TMLI: Total marrow and lymphatic irradiation
TPS: Treatment planning system
